# Adipsin Is Associated with Multiple Sclerosis: A Follow-Up Study of Adipokines

**DOI:** 10.1155/2015/371734

**Published:** 2015-11-08

**Authors:** Renuka Natarajan, Sanna Hagman, Mari Hämälainen, Tiina Leppänen, Prasun Dastidar, Eeva Moilanen, Irina Elovaara

**Affiliations:** ^1^Neuroimmunology Unit, Medical School, University of Tampere, Tampere University Hospital, Biokatu 10, 33520 Tampere, Finland; ^2^The Immunopharmacology Research Group, University of Tampere, School of Medicine and Tampere University Hospital, Medisiinarinkatu 3, 33520 Tampere, Finland; ^3^Department of Radiology, Medical Imaging Centre, Tampere University Hospital, Teiskontie 35, 33520 Tampere, Finland; ^4^Department of Neurology, Tampere University Hospital, Teiskontie 35, 33520 Tampere, Finland

## Abstract

*Background and Objective*. The role of adipokines in regulation of immune responses has been recognized, but very little is known about their impact on multiple sclerosis (MS). In this study, we analysed whether the major adipokines are differentially expressed in plasma of patients with different MS subtypes and clinically isolated syndrome (CIS) and explored their association with major disease characteristics. *Methods*. The levels of adiponectin, adipsin, leptin, and resistin in the plasma of 80 patients with different subtypes of MS and CIS were followed up annually over the two years. The data obtained were correlated with disease activity, EDSS and volumes of T1-weighted lesions (T1-LV), and fluid attenuation inversion recovery lesions (FLAIR-LV) on MRI. *Results*. In MS group, a correlation was found between the level of adipsin and EDSS score at baseline (*r* = 0.506, *p* < 0.001). In RRMS, the levels of adipsin correlated with EDSS scores (*r* = 0.542, *p* = 0.002), T1-LV (*r* = 0.410, *p* = 0.034), and FLAIR-LV (*r* = 0.601, *p* = 0.0001) at baseline and an increase in the T1-LV over the follow-up (*r* = 0.582, *p* = 0.003). Associations with other adipokines were not detected. *Conclusion*. Our exploratory study provides novel insights on the impact of adipokines in MS and suggests that adipsin exerts predictive potential as a biomarker of neurodegeneration.

## 1. Introduction

Multiple sclerosis (MS) is an autoimmune demyelinating disease of the central nervous system (CNS) mediated by the transendothelial migration of activated T helper 1 (Th1) and Th17 lymphocytes into the brain tissue where they trigger the destructive inflammatory cascade resulting in the accumulation of inflammatory infiltrates, demyelination, axonal loss, and gliosis [1, 2]. The damage to neural tissue is induced by various effector mechanisms and substances such as macrophage phagocytosis, secretion of inflammatory cytokines, chemokines and antibodies, complement activation, mitochondrial dysfunction, release of cytotoxic proteases, and products of oxidative stress and excitotoxicity that all together contribute to the development of neurological worsening [1].

Increased risk of MS in subjects with obesity during adolescence and early adulthood has been recently reported [3, 4]. It has been considered that such risk is explained by modulatory effect of adipose tissue on inflammatory responses in obese subjects. Indeed, adipose tissue is recognized as an endocrine organ that secretes multiple cytokine-like hormones, adipokines that are involved in regulation of multiple physiological functions including inflammation [5, 6]. Although dysregulation of adipokines during obesity and in autoimmune diseases has been recognized, only very little is known about their role in MS [5]. The best-known adipokines are the proinflammatory leptin, adipsin, resistin and visfatin, and the anti-inflammatory adiponectin, omentin-1, and apelin [5, 7]. Up to now, most studies in MS have been focused on leptin that was found to be increased in blood and cerebrospinal fluid (CSF) of MS patients, but contrasting results have also been reported [8–11]. In these studies, leptin correlated negatively with the number of regulatory T cells [11], but associations to clinical parameters were not reported. The levels of visfatin and resistin in sera of MS patients were found to be increased, while the levels of adiponectin were downregulated [9, 12–14]. Until now only one study analysed several adipokines in a cohort of patients including different subtypes of MS [14]. According to this study, elevated levels of visfatin and decreased levels of leptin were found in patients with relapsing-remitting MS (RRMS), but association with clinical parameters was not detected.

Due to the sparse knowledge on the association of adipokines with clinical characteristics of MS, the purpose of this two-year prospective follow-up study was to assess whether the levels of adiponectin, adipsin, leptin, and resistin in plasma of MS patients are associated with clinical phenotypes, inflammatory disease activity, neurological disability, and the volumes of T1-weighted and fluid attenuation inversion recovery (FLAIR) lesions on magnetic resonance imaging (MRI).

## 2. Patients and Methods

### 2.1. Subjects

This two-year prospective follow-up study included altogether 80 subjects of whom 65 had clinically definite MS (CDMS) according to the revised McDonald Criteria [15] and 15 had clinically isolated syndrome (CIS) [16]. The CDMS group included 34 patients with RRMS, 15 patients with secondary progressive MS (SPMS), and 16 subjects with primary progressive MS (PPMS). CIS patients were defined as patients who had had their first demyelinating neurologic event suggestive of MS [16]. All patients underwent annual neurological examinations from baseline up to two years. The blood was drawn on the same day as the neurological examination. The clinical evaluation included the determination of body mass index (BMI, kg/m^2^), prestudy disease activity (number of relapses two years before the study), number of relapses over the two-year follow-up, and Expanded Disability Status Scale (EDSS) score [17] at the baseline and the end of the follow-up as summarized in Table 1. Patients who were pregnant or suffering from any other clinically significant diseases were excluded. The study was approved by the Ethics Committee of Tampere University Hospital and all subjects gave informed consent.

### 2.2. MRI Image Segmentation and Volumetric Analysis

All patients underwent MRI examination at baseline and at the end of follow-up period. All examinations were performed on a 1.5 Tesla MRI Unit (Siemens Avanto, Erlangen, Germany). The MRI protocol included a T1-weighted header followed by axial T1-weighted magnetization prepared rapid gradient echo (MP-RAGE) and T2-weighted turbo spin-echo (TSE), FLAIR, magnetization transfer contrasts (MTC), diffusion weighted imaging (DWI), and gadolinium enhanced T1-weighted MP-RAGE sequences. T1-weighted MP-RAGE, FLAIR, and T2-weighted TSE images were used for volumetric analysis. For MP-RAGE, the imaging parameters were as follows: repetition time (TR) = 1160 ms; echo time (TE) = 4.24 ms; inversion time (TI) = 600 ms; slice thickness = 0.9 mm; in-plane resolution = 0.45*∗*0.45 mm. In FLAIR images, the following parameters were used: TR = 8500 ms; TE = 100 ms; TI = 2500 ms; slice thickness = 5.0 mm; in-plane resolution = 0.45*∗*0.45 mm. In TSE, the following imaging scheme was used: TR = 750 ms; TE = 115 ms; slice thickness = 3.0 mm; in-plane resolution = 0.90*∗*0.90 mm. Volumetric segmentation of plaques in the brain was performed using semiautomatic software Anatomatic operating in a PC/Window 95 environment [18, 19] and the images were analysed blind.

### 2.3. Determination of Adipokines

Venous blood was collected for the assessment of plasma levels of adiponectin, adipsin, leptin, and resistin. Blood containing tubes were centrifuged for 15 min at 1600 ×g. Plasma was separated from the blood, aliquoted, and stored at −70°C until use. Adipokines were determined by enzyme-linked immunoassay (ELISA) using commercial reagents according to the manufacturers' instructions (DuoSet ELISA, R&D Systems Europe Ltd., Abingdon, UK). The respective detection limits and interassay coefficients of variation were 15.6 ng/L and 2.0% for adiponectin, 4.0 ng/mL and 3.8% for adipsin, 15.6 ng/L and 3.9% for leptin, and 15.6 ng/L and 4.0% for resistin.

### 2.4. Statistical Analysis

Statistical analyses were performed with SPSS version 18.0 (SPSS Inc., Chicago, IL, USA). A *p* value less than 0.05 was considered significant in all analyses. Mann-Whitney *U* test was used to analyse the differences in clinical parameters and MRI volumes between the subtypes. Wilcoxon signed-rank test was used to analyse the intraindividual changes in the volumes of MRI at each time point.

For comparison of the adipokines levels in different subtypes, repeated measures of ANOVA followed by Bonferroni correction for multiple comparisons were used. For each outcome, the analyses were also adjusted for age and gender. Pearson's correlation coefficient was used to explore the relationship between the levels of adipokines with BMI or age. The associations of adipsin levels with EDSS scores and the volumes of T1-weighted and FLAIR lesions were studied by linear regression model by adjusting for age, gender, and disease subtype. Logistic regression model was used to study the association between adipokines and disease activity. The differences in the adipokines levels between genders were studied by Mann-Whitney *U* test.

## 3. Results

### 3.1. Clinical and MRI Follow-Up

#### 3.1.1. Clinical Data

The demographic and two-year clinical follow-up data of the subjects are summarized in Table 1. As expected, the patients in the SPMS and PPMS groups had longer disease duration and were older than the patients with RRMS or CIS (*p* < 0.05). The EDSS scores were lower in CIS and RRMS than in other MS subtypes (*p* < 0.05), while no differences were found between SPMS and PPMS. There were no differences in BMI between any of the MS subtypes and CIS (*p* > 0.05).

At the end of the follow-up, the EDSS score was increased in 27% (*n* = 17/65) of CDMS patients: (21% RRMS, 40% SPMS, and 25% PPMS). Two years before study entry, half of RRMS patients were relapse-free, 12% had one relapse, and the remaining 38% of subjects had 2–5 relapses. At the end of the follow-up, 68% of RRMS patients were relapse-free, 15% of patients had one relapse, and the remaining 17% of subjects had 2–5 relapses. The majority of RRMS patients were treated with immunomodulatory drugs (53% interferon-beta (IFN-*β*), 6% glatiramer acetate, and 3% mitoxantrone). At the end of follow-up, 35% of patients were treated with IFN-*β*, 26% of patients with copaxone, and 3% of patients with natalizumab.

The baseline EDSS score of CIS patients was 0 except for two subjects having score 1. Over the two-year period, 7 out of 15 CIS patients converted to CDMS. All converted patients had elevated IgG index and OCBs in their CSF.

#### 3.1.2. Volumes of T1-Weighted and FLAIR Lesions

The volumes of MS plaques were determined in the 75 MS and CIS patients at the baseline and after one year (Table 2). As expected, the baseline volumes of T1-weighted and FLAIR lesions were lowest in the CIS group (*p* < 0.01). Baseline comparison between the MS subtypes showed higher FLAIR and T1 lesion volumes in SPMS than in PPMS or RRMS (*p* < 0.05). Over the follow-up, the volumes of these lesions increased in all studied groups (*p* < 0.05).

### 3.2. Levels of Adipokines in MS Subtypes during the Two-Year Follow-Up

**Figure 1:**
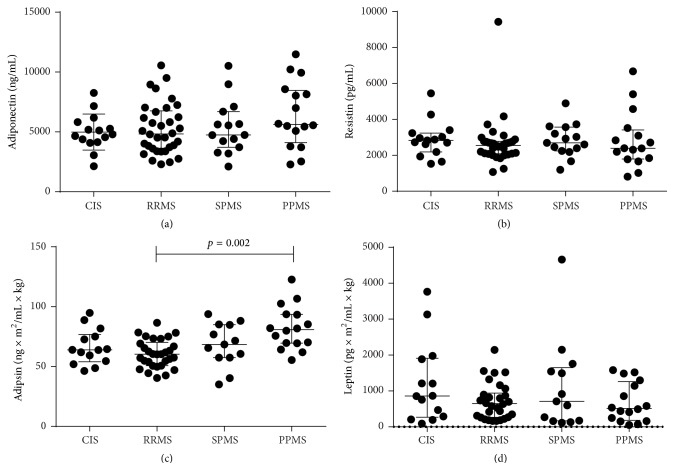
Scatter plot showing the baseline levels of Adiponectin (A), Resistin (B), BMI-adjusted Adipsin (C) and BMI-adjusted Leptin (D) in MS and CIS. The bars indicate the median and interquartile range.

Correlation analyses assessing associations of adipokines with BMI in CDMS group showed correlations with levels of adipsin (*r* = 0.277, *p* = 0.018) and leptin (*r* = 0.491, *p* < 0.0001) but not with adiponectin (*r* = −0.132, *p* = 0.267) or resistin (*r* = −0.071, *p* = 0.551). Due to observed correlations with adipsin and leptin, these adipokines were adjusted by dividing their concentrations by BMI. To assess the differences in the adipokines levels between different MS subtypes, repeated measures of ANOVA adjusting for age and gender were used. It appeared that over the two years the levels of adipokines in different groups remained stable (Table 3). The levels of BMI-adjusted adipsin in RRMS patients were lower than those in subjects with PPMS throughout the whole follow-up period (Table 3) (*p* = 0.002). After controlling for age alone, the difference in the adipsin levels between the groups was still statistically significant (*p* = 0.037 adjusted), while after adjusting simultaneously for age and gender only a trend toward statistical significance was found (*p* = 0.057). Other adipokines levels did not differ between the subtypes.  Figure 1 illustrates the baseline distribution of the adipokines levels in patients with different subtypes. Notably, the levels of BMI-adjusted adipsin in treated and untreated RRMS patients were decreased in comparison to PPMS, but no differences were found between these RRMS groups (Supplementary Figure 1 in Supplementary Material available online at http://dx.doi.org/10.1155/2015/371734). Likewise, the levels of adipokines were of the similar magnitude in converted and nonconverted patients with CIS.

The influence of gender on secretion of adipokines was studied by comparing the baseline levels in men and women. It appeared that in CDMS group the levels of leptin (869.6 (536.9–1504.9) versus 242.3 (152.9–441.3) pg × m^2^/mL × kg, *p* < 0.001) and adiponectin (5540.9 (4197.4–8036.1) versus 3808.0 (3178.7–5545.4)  ng/mL, *p* = 0.010, median (interquartile range)) were higher in women. In CDMS, also the age correlated with the levels of BMI-adjusted adipsin (*r* = 0.491, *p* < 0.001).

### 3.3. Association of Adipokines with Clinical and MRI Measures

**Figure 2:**
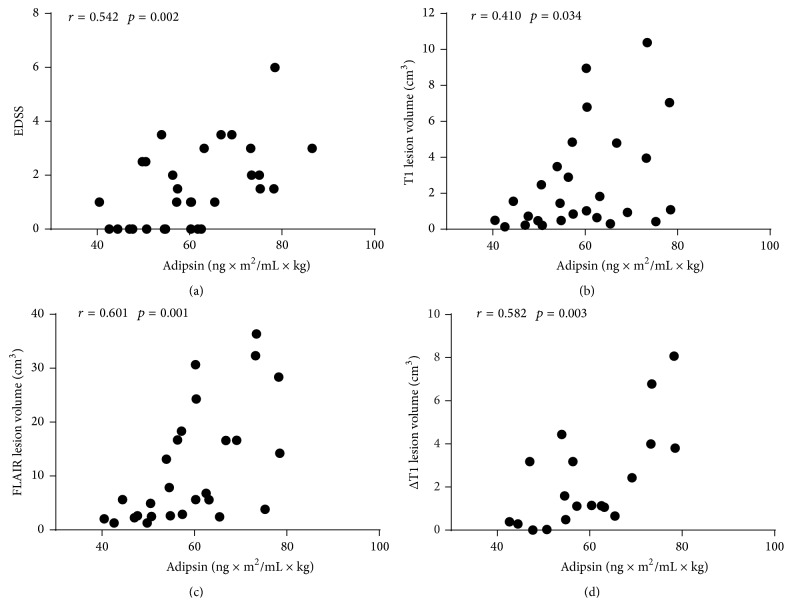
Associations between baseline BMI-adjusted adipsin and EDSS score (a), the volumes of T1 lesions (b), FLAIR lesions (c), and changes of T1 lesion volumes over the follow-up (d) in RRMS patients.

Association of adipokines levels with baseline EDSS score and the volumes of FLAIR- or T1-weighted lesions as well as the change of their volumes over the follow-up were studied by linear regression model adjusting for age, gender, and disease subtype. In the CDMS group, the analyses among the adipokines showed a positive correlation between the baseline BMI-adjusted adipsin and EDSS scores (*r* = 0.506, *p* < 0.001), and such associations were also observed after adjusting for age alone (*r* = 0.387,  *p* = 0.003), for age and gender (*r* = 0.376, *p* = 0.004), or for combination of age, gender, and disease subtype (*r* = 0.280, *p* = 0.036). According to subtype analysis, in RRMS group the correlation was even stronger (*r* = 0.542, *p* = 0.002; Figure 2(a)). Similar associations were observed after adjusting for age (*r* = 0.570, *p* = 0.001) or age and gender (*r* = 0.569,  *p* = 0.002). Over the two years, the EDSS score increased in 27% of CDMS patients (*n* = 17/65) (21% RRMS, 40% SPMS, and 25% PPMS), but the levels of adipokines did not associate with this change.

In the CDMS group, the levels of adipokines did not associate with the volumes of FLAIR- or T1-weighted lesions or the change of their volumes over the follow-up (*p* > 0.05). However, according to subgroup analysis, in RRMS correlations were found between the baseline levels of BMI-adjusted adipsin and the volumes of T1-weighted (*r* = 0.410, *p* = 0.034; Figure 2(b)) and FLAIR (*r* = 0.601, *p* = 0.0001; Figure 2(c)) lesions or the changes of T1 lesion volumes over the follow-up (*r* = 0.582, *p* = 0.003; Figure 2(d)). After adjusting for age and gender, RRMS group still showed positive correlations in these measures indicating that age and gender did not have an impact on these correlations (Table 4).

**Figure 3:**
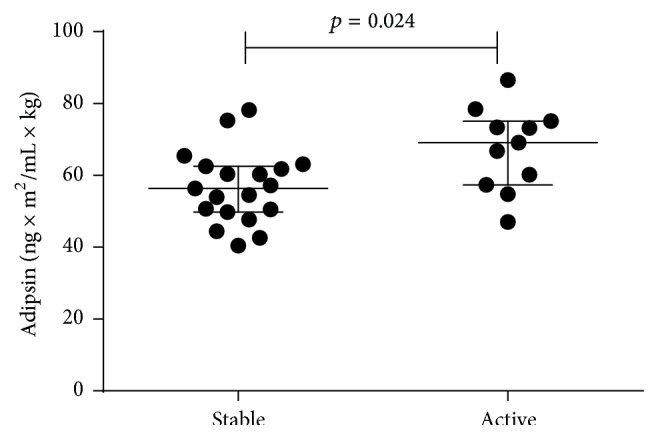
Scatter plot showing the levels of adipsin in stable and active RRMS patients. The bars indicate the median and interquartile range.

We next analysed whether the levels of adipokines are associated with clinical or MRI disease activity before study entry and over the follow-up period. Baseline clinical disease activity was determined by the presence of at least 2 relapses during 2 years before study entry and baseline MRI activity by presence of at least one Gd-enhancing lesion. The disease activity on MRI over the follow-up was assessed based on the presence of at least one Gd-enhancing lesion or new T2 lesion. At the study entry, the presence of higher clinical disease activity (*n* = 13, at least 2 relapses/2 years before baseline) was associated with higher levels of BMI-adjusted adipsin in comparison to patients with stable disease course (*n* = 21, 0-1 relapses/2 years before baseline) (Figure 3). However, no associations were found over the follow-up. Regarding the MRI activity, half of the patients (18/34) showed MRI activity according to defined criteria (presence of Gd-enhancing lesion or new T2 lesion over the follow-up period), but association between adipokines levels and MRI activity was not found.

## 4. Discussion

Currently very little is known about the impact of adipokines on MS. This exploratory study assessed the ability of best-known adipokines to discriminate between MS subtypes and their potential to depict inflammatory activity and neurological deterioration in MS. A correlation between the baseline levels of adipsin and EDSS scores detected in whole MS and RRMS cohorts suggests an involvement of adipsin in pathophysiology of MS. Such interpretation is further supported by the correlations detected between the baseline adipsin and the volumes of T1-weighted and FLAIR lesions as well as the change of such lesion volumes over the follow-up seen in RRMS group.

Adipsin (complement factor D) is a key enzyme involved in the activation of alternative pathway of complement activation and is primarily secreted from adipocytes and monocytes/macrophages in human subjects [20]. Its role in the pathogenesis of MS has not been studied, but the immunohistochemical studies have demonstrated the presence of other complement components within the lesion in normal appearing white matter (NAWM) and cortical areas suggesting involvement of complement proteins in MS [21, 22]. Complement components of the classical and alternative pathway including C3, C4, C5, C9, terminals complement complex (TCC), complement receptor, and factors B, I, and H [23–31] have been previously analysed in sera and CSF of MS patients [23–31]. These studies have showed the positive correlation between CSF levels of C3, C9, and TCC and EDSS scores [30, 32, 33].

The observed association of baseline adipsin with neurological disability expressed by EDSS score in whole MS and RRMS cohorts suggests a role of adipsin in accumulation of neurological disability. Moreover, in RRMS at baseline an association between the adipsin and the volumes of T1-weighted lesions as well as their increase over the follow-up suggests predictive potential of adipsin as a biomarker of neurodegeneration. According to statistical analyses, age and gender did not influence these results. The absence of evolution of adipokines levels over the follow-up is most likely explained by relatively stable clinical disease course in most of our patients. However, an increase of the volumes of T1 and FLAIR lesions seen by MRI is consistent with worsening of MS even during the relatively short follow-up in this study. In parallel, the presence of higher adipsin in a subgroup of patients with more active RRMS (≥2 relapses/2 years before baseline) together with a positive correlation between the baseline adipsin and the volumes of FLAIR lesions in whole RRMS group suggests an involvement of adipsin also in inflammatory disease activity. Taken together, according to these observations adipsin is a neuroinflammation-promoting molecule that facilitates neurological deterioration and underlying neurodegeneration. It is noteworthy that inflammation-promoting activity of alternative complement pathway on adaptive immune responses has been recently reported also by other investigators [34, 35]. According to these studies, anaphylatoxins especially produced during the activation of alternative pathway may trigger inflammation and chemotaxis [34], although the role of complement in the adaptive immune responses to induce the T cell activation and proliferation has also been proposed [35].

The presence of decreased adipsin in RRMS patients in comparison to those with PPMS is most likely related to different pathological mechanisms in these MS subtypes. The early phase of RRMS is characterized predominantly by inflammatory events initiated by activation and differentiation of myelin specific CD4+ T cells into Th1 and Th17 cells and their transmigration from periphery to CNS eventually resulting in demyelination and axonal loss [36]. During the transition to more advanced stages like SPMS, BBB becomes less permeable leading to diminished entry of peripheral immune cells and their products into CNS [37]. Recent pathologic studies have showed that progressive subtypes are characterized by the widespread diffuse inflammation with slowly expanding lesions, abundant cortical lesions, and lymphocyte infiltration and microglia activation in the NAWM [38]. The elevated levels of adipsin in our PPMS patients most likely reflect peripheral immune activation and do not associate with ongoing focal CNS changes seen on MRI. Notably, recently the other member of complement pathway, that is, complement factor H, was found to be elevated in sera of patients with progressive MS but not in RRMS or healthy controls [39]. Together the available data suggest that elevated levels of adipsin in patients with progressive MS reflect ongoing peripheral immune activation.

Interestingly, this study revealed positive correlations between the BMI and the levels of leptin and adipsin. These observations support the hypothesis of close interaction between the adipose tissue and immune system in regulation of inflammatory responses [5]. In addition, the presence of higher levels of leptin and adiponectin in women indicates the presence of gender-specific association to secretion of these adipokines. Parallel results in MS and healthy subjects have been reported also by others [8, 40, 41].

## 5. Conclusions

This study showed an association of adipsin to neurological disability and focal changes on MRI in MS thus suggesting that dysregulation of alternate complement pathway may have an impact on MS disease course. The data suggest that adipsin exerts an inflammation-promoting effect and facilitates the development of neurodegenerative changes. The predictive potential of adipsin as a biomarker of neurodegeneration needs to be evaluated in further studies.

## Supplementary Material

Supplementary Figure 1: The levels of Adiponectin (A), Resistin (B), Adipsin (C) and Leptin (D) in MS patients including treated and untreated patients.

## Figures and Tables

**Table 1:** Demographic and clinical characteristics of patients with different MS phenotypes.

	RRMS *n* = 34	SPMS *n* = 15	PPMS *n* = 16	CIS *n* = 15
Gender F/M^a^	24/10	10/5	9/7	13/2
Age (years)^b^	37.6 ± 9.2 (20–51)	49.3 ± 10.0 (32–63)	58.1 ± 8.5 (40–70)	35.6 ± 7.9 (24–51)
BMI (kg/m^2^)^b^	24.9 ± 4.0 (19.7–35.4)	26.2 ± 5.0 (15.8–33.3)	24.6 ± 3.4 (19.8–31.5)	25.3 ± 3.2 (21.6–31.2)
Disease duration from first symptoms (years)^b^	8.2 ± 7.2 (0.7–29.6)	19.8 ± 7.8 (6.3–34.3)	18.9 ± 9.6 (2.4–43.0)	3.0 ± 2.5 (0.5–8.9)
Disease duration from diagnosis (years)^b^	4.2 ± 4.1 (0.0–13.7)	12.9 ± 9.0 (2.2–32.4)	13.1 ± 8.4 (1.5–27.2)	NA
EDSS at baseline^b^	1.4 ± 1.5 (0.0–6.0)	5.2 ± 1.6 (2.5–7.5)	4.7 ± 2.2 (1.0–7.0)	0.1 ± 0.3 (0.0–1.0)
EDSS at end of the follow-up^b^	1.5 ± 1.6 (0.0–6.0)	5.5 ± 1.6 (2.5–8.0)	4.8 ± 2.1 (1.5–7.0)	0.1 ± 0.4 (0.0–1.0)
EDSS worsening during follow-up^c^	7 (21%)	6 (40%)	4 (25%)	1 (7%)
Prestudy disease activity^b,d^	1.2 ± 1.4 (0–5)	0.2 ± 0.6 (0–2)	0.0 ± 0.0 (0-0)	0.7 ± 0.6 (0–2)
Number of relapses over the follow-up^b^	0.6 ± 1.1 (0–5)	0.4 ± 0.7 (0–2)	0.0 ± 0.0 (0-0)	0.1 ± 0.3 (0-1)
Treatment (NT/IFN/CO/MX)^a^	12/18/3/1	15/0/0/0	16/0/0/0	15/0/0/0

RRMS: relapsing-remitting MS, SPMS: secondary progressive MS, PPMS: primary progressive MS, CIS: clinically isolated syndrome, BMI: body mass index, EDSS: expanded disability status scale, NT: no treatment, IFN: interferon, CO: copaxone, MX: mitoxantrone, and NA: not applicable.

^a^Number of patients.

^b^Mean ± SD (range).

^c^Number of patients (percent).

^d^Number of relapses in the 2 years before baseline.

**Table 2:** Volumes of T1 and FLAIR lesions at baseline and follow-up (median (interquartile range)).

	T1 lesions (cm^3^)			FLAIR lesions (cm^3^)		
	BL	1-YR	*p* value^a^	BL	1-YR	*p* value^a^
CIS	0.4 (0.0–0.6)	0.3 (0.2–0.8)	0.046	1.0 (0.3–2.2)	1.3 (0.5–3.1)	0.001
RRMS	1.4 (0.5–3.7)^*∗*,#^	3.0 (0.8–6.0)	0.001	6.3 (2.6–17.5)^*∗*,#^	14.0 (6.0–24.8)	0.00002
SPMS	5.8 (2.2–9.4)^*∗*^	6.5 (3.7–18.6)	0.041	18.4 (11.6–30.1)^*∗*^	29.3 (24.8–40.2)	0.004
PPMS	0.8 (0.6–2.8)^*∗*,#^	2.0 (0.9–4.7)	0.004	5.3 (3.1–10.4)^*∗*,#^	9.7 (6.8–15.1)	0.0004

RRMS: relapsing-remitting MS, SPMS: secondary progressive MS, PPMS: primary progressive MS, CIS: clinically isolated syndrome, and BL: baseline.

^a^The intraindividual changes in the volumes of MRI over the follow-up period, Wilcoxon signed-rank test.

^*∗*^Compared to CIS group, Mann-Whitney *U* test, *p* < 0.01.

^#^Compared to SPMS, Mann-Whitney *U* test, *p* < 0.05.

**Table 3:** The levels of adipokines in MS subtypes and CIS over follow-up period (median (interquartile range)).

	Baseline	One year	Two years
Adiponectin (ng/mL)			
CIS RRMS SPMS PPMS	4720.6 (4124.4–5407.7) 4517.3 (3380.6–6417.2) 4748.4 (3977.9–6379.9) 5618.7 (4111.5–8467.1)	5131.0 (4239.4–5880.3) 4695.7 (3452.4–7270.5) 5314.8 (4289.4–7494.9) 5855.8 (4622.6–9948.3)	5149.7 (4789.0–6812.0) 5012.7 (3222.8–7043.1) 6062.3 (4706.5–7871.2) 5278.5 (3761.6–8779.1)

Adipsin (ng × m^2^/mL × kg)^a^			
CIS RRMS SPMS PPMS	63.9 (53.9–76.8) 60.3 (52.3–71.2)^*∗*,#^ 68.4 (57.4–85.0) 80.8 (69.5–93.6)	66.4 (56.9–83.5) 62.4 (55.1–71.3)^*∗*,#^ 70.3 (63.3–81.7) 75.9 (67.1–94.6)	66.7 (59.7–74.4) 61.5 (56.2–69.6)^*∗*,#^ 74.1 (57.7–89.6) 82.5 (66.1–95.0)

Leptin (pg × m^2^/mL × kg)^b^			
CIS RRMS SPMS PPMS	861.0 (269.8–1908.4) 662.4 (288.1–981.4) 711.1 (166.8–1649.7) 515.2 (182.6–1260.2)	694.9 (240.1–1238.7) 572.4 (286.7–1159.3) 420.0 (157.5–1496.2) 603.3 (203.5–1092.2)	754.5 (424.2–1346.5) 572.0 (321.4–1032.9) 953.2 (287.9–1822.7) 479.3 (255.6–776.1)

Resistin (pg/mL)			
CIS RRMS SPMS PPMS	2851.5 (2509.1–3280.5) 2505.2 (2085.0–2751.0) 2605.4 (2216.0–3388.0) 2392.5 (1794.9–3411.5)	2946.6 (2367.6–3093.8) 2402.1 (2064.9–2869.3) 2649.9 (2358.5–3146.4) 2371.2 (1663.5–3354.6)	2769.5 (2299.2–3072.8) 2315.9 (1946.9–2796.2) 2469.0 (2284.6–2975.3) 2445.4 (1711.1–3349.5)

^a^BMI-adjusted adipsin levels (ng × m^2^/mL × kg).

^b^BMI-adjusted leptin levels (pg × m^2^/mL × kg).

^*∗*^Comparison of PPMS *p* < 0.01.

^#^Comparison of PPMS after adjusting for age *p* < 0.05.

**Table 4:** Observed associations between the levels of adipsin and clinical and MRI parameters (Pearson's correlation coefficient (*p* value)).

Parameters		Basic model	Adjusted for age	Adjusted for age and gender	Adjusted for age, gender, and subtype
EDSS	CDMS	0.506 (<0.0001)	0.387 (0.003)	0.376 (0.004)	0.280 (0.036)
	RRMS	0.542 (0.002)	0.570 (0.001)	0.569 (0.002)	—
T1 lesion volume	RRMS	0.410 (0.034)	0.402 (0.042)	0.407 (0.043)	—
FLAIR lesion volume	RRMS	0.601 (0.001)	0.596 (0.001)	0.596 (0.002)	—
ΔT1 lesion volume	RRMS	0.582 (0.003)	0.582 (0.004)	0.583 (0.004)	—

CDMS: clinically definite MS, RRMS: relapsing-remitting MS, EDSS: expanded disability status scale, and FLAIR: fluid attenuation inversion recovery.

## References

[1] Friese M. A., Schattling B., Fugger L. (2014). Mechanisms of neurodegeneration and axonal dysfunction in multiple sclerosis. *Nature Reviews Neurology*.

[2] Ellwardt E., Zipp F. (2014). Molecular mechanisms linking neuroinflammation and neurodegeneration in MS. *Experimental Neurology*.

[3] Munger K. L., Chitnis T., Ascherio A. (2009). Body size and risk of MS in two cohorts of US women. *Neurology*.

[4] Hedström A. K., Olsson T., Alfredsson L. (2012). High body mass index before age 20 is associated with increased risk for multiple sclerosis in both men and women. *Multiple Sclerosis*.

[5] Versini M., Jeandel P.-Y., Rosenthal E., Shoenfeld Y. (2014). Obesity in autoimmune diseases: not a passive bystander. *Autoimmunity Reviews*.

[6] Exley M. A., Hand L., O'Shea D., Lynch L. (2014). Interplay between the immune system and adipose tissue in obesity. *Journal of Endocrinology*.

[7] Aguilar-Valles A., Inoue W., Rummel C., Luheshi G. N. (2015). Obesity, adipokines and neuroinflammation. *Neuropharmacology*.

[8] Rotondi M., Batocchi A. P., Coperchini F. (2013). Severe disability in patients with relapsing-remitting multiple sclerosis is associated with profound changes in the regulation of leptin secretion. *NeuroImmunoModulation*.

[9] Kraszula Ł., Jasińska A., Eusebio M.-O., Kuna P., Głabiński A., Pietruczuk M. (2012). Evaluation of the relationship between leptin, resistin, adiponectin and natural regulatory T cells in relapsing-remitting multiple sclerosis. *Neurologia i Neurochirurgia Polska*.

[10] Batocchi A. P., Rotondi M., Caggiula M. (2003). Leptin as a marker of multiple sclerosis activity in patients treated with interferon-beta. *Journal of Neuroimmunology*.

[11] Matarese G., Carrieri P. B., La Cava A. (2005). Leptin increase in multiple sclerosis associates with reduced number of CD4^+^CD25^+^ regulatory T cells. *Proceedings of the National Academy of Sciences of the United States of America*.

[12] Musabak U., Demirkaya S., Genç G., Ilikci R. S., Odabasi Z. (2010). Serum adiponectin, TNF-*α*, IL-12p70, and IL-13 levels in multiple sclerosis and the effects of different therapy regimens. *NeuroImmunomodulation*.

[13] Hietaharju A., Kuusisto H., Nieminen R., Vuolteenaho K., Elovaara I., Moilanen E. (2010). Elevated cerebrospinal fluid adiponectin and adipsin levels in patients with multiple sclerosis: a Finnish co-twin study. *European Journal of Neurology*.

[14] Emamgholipour S., Eshaghi S. M., Hossein-Nezhad A., Mirzaei K., Maghbooli Z., Sahraian M. A. (2013). Adipocytokine profile, cytokine levels and FOXP3 expression in multiple sclerosis: a possible link to susceptibility and clinical course of disease. *PLoS ONE*.

[15] Polman C. H., Reingold S. C., Banwell B. (2011). Diagnostic criteria for multiple sclerosis: 2010 revisions to the McDonald criteria. *Annals of Neurology*.

[16] Polman C. H., Reingold S. C., Edan G. (2005). Diagnostic criteria for multiple sclerosis: 2005 revisions to the ‘McDonald Criteria’. *Annals of Neurology*.

[17] Kurtzke J. F. (1983). Rating neurologic impairment in multiple sclerosis: an expanded disability status scale (EDSS). *Neurology*.

[18] Heinonen T., Dastidar P., Eskola H., Frey H., Ryymin P., Laasonen E. (1998). Applicability of semi-automatic segmentation for volumetric analysis of brain lesions. *Journal of Medical Engineering and Technology*.

[19] Heinonen T., Dastidar P., Kauppinen P., Malmivuo J., Eskola H. (1998). Semi-automatic tool for segmentation and volumetric analysis of medical images. *Medical & Biological Engineering and Computing*.

[20] White R. T., Damm D., Hancock N. (1992). Human adipsin is identical to complement factor D and is expressed at high levels in adipose tissue. *The Journal of Biological Chemistry*.

[21] Ingram G., Loveless S., Howell O. W. (2014). Complement activation in multiple sclerosis plaques: an immunohistochemical analysis. *Acta Neuropathologica Communications*.

[22] Zhou W. (2012). The new face of anaphylatoxins in immune regulation. *Immunobiology*.

[23] Halawa I., Lolli F., Link H. (1989). Terminal component of complement C9 in CSF and plasma of patients with MS and aseptic meningitis. *Acta Neurologica Scandinavica*.

[24] Jans H., Heltberg A., Zeeberg I., Kristensen J. H., Fog T., Raun N. E. (1984). Immune complexes and the complement factors C4 and C3 in cerebrospinal fluid and serum from patients with chronic progressive multiple sclerosis. *Acta Neurologica Scandinavica*.

[25] Jongen P. J. H., Doesburg W. H., Ibrahim-Stappers J. L. M., Lemmens W. A. J. G., Hommes O. R., Lamers K. J. B. (2000). Cerebrospinal fluid C3 and C4 indexes in immunological disorders of the central nervous system. *Acta Neurologica Scandinavica*.

[26] Mollnes T. E., Vandvik B., Lea T., Vartdal F. (1987). Intrathecal complement activation in neurological diseases evaluated by analysis of the terminal complement complex. *Journal of the Neurological Sciences*.

[27] Morgan B. P., Campbell A. K., Compston D. A. S. (1984). Terminal component of complement (C9) in cerebrospinal fluid of patients with multiple sclerosis. *The Lancet*.

[28] Rodriguez M., Wynn D. R., Kimlinger T. K., Katzmann J. A. (1990). Terminal component of complement (C9) in the cerebrospinal fluid of patients with multiple sclerosis and neurologic controls. *Neurology*.

[29] Sanders M. E., Koski C. L., Robbins D., Shin M. L., Frank M. M., Joiner K. A. (1986). Activated terminal complement in cerebrospinal fluid in Guillain-Barre syndrome and multiple sclerosis. *Journal of Immunology*.

[30] Sellebjerg F., Jaliashvili I., Christiansen M., Garred P. (1998). Intrathecal activation of the complement system and disability in multiple sclerosis. *Journal of the Neurological Sciences*.

[31] Ingram G., Hakobyan S., Hirst C. L. (2010). Complement regulator factor H as a serum biomarker of multiple sclerosis disease state. *Brain*.

[32] Ingram G., Hakobyan S., Hirst C. L. (2012). Systemic complement profiling in multiple sclerosis as a biomarker of disease state. *Multiple Sclerosis*.

[33] Aeinehband S., Lindblom R. P., Al Nimer F. (2015). Complement component C3 and butyrylcholinesterase activity are associated with neurodegeneration and clinical disability in multiple sclerosis. *PLoS ONE*.

[34] Ingram G., Hakobyan S., Robertson N. P., Morgan B. P. (2009). Complement in multiple sclerosis: its role in disease and potential as a biomarker. *Clinical and Experimental Immunology*.

[35] Kwan W.-H., van der Touw W., Heeger P. S. (2012). Complement regulation of T cell immunity. *Immunologic Research*.

[36] Yadav S. K., Mindur J. E., Ito K., Dhib-Jalbut S. (2015). Advances in the immunopathogenesis of multiple sclerosis. *Current Opinion in Neurology*.

[37] Revesz T., Kidd D., Thompson A. J., Barnard R. O., McDonald W. I. (1994). A comparison of the pathology of primary and secondary progressive multiple sclerosis. *Brain*.

[38] Haugen M., Frederiksen J. L., Degn M. (2014). B cell follicle-like structures in multiple sclerosis-with focus on the role of B cell activating factor. *Journal of Neuroimmunology*.

[39] Ingram G., Hakobyan S., Loveless S., Robertson N., Morgan B. P. (2011). Complement regulator factor H in multiple sclerosis. *Journal of Cellular Biochemistry*.

[40] Evangelopoulos M. E., Koutsis G., Markianos M. (2014). Serum leptin levels in treatment-naive patients with clinically isolated syndrome or relapsing-remitting multiple sclerosis. *Autoimmune Diseases*.

[41] Saad M. F., Damani S., Gingerich R. L. (1997). Sexual dimorphism in plasma leptin concentration. *The Journal of Clinical Endocrinology & Metabolism*.

