# Supplementary material for: Adipsin Is Associated with Multiple Sclerosis: A Follow-Up Study of Adipokines
Source: Mult Scler Int. 2015 Nov 8;2015:371734. doi: 10.1155/2015/371734 (PMC4655075; doi:10.1155/2015/371734)

Supplementary figure 1. The levels of Adiponectin (A), Resistin (B), Adipsin (C) and Leptin (D) in MS patients including treated and untreated patients.

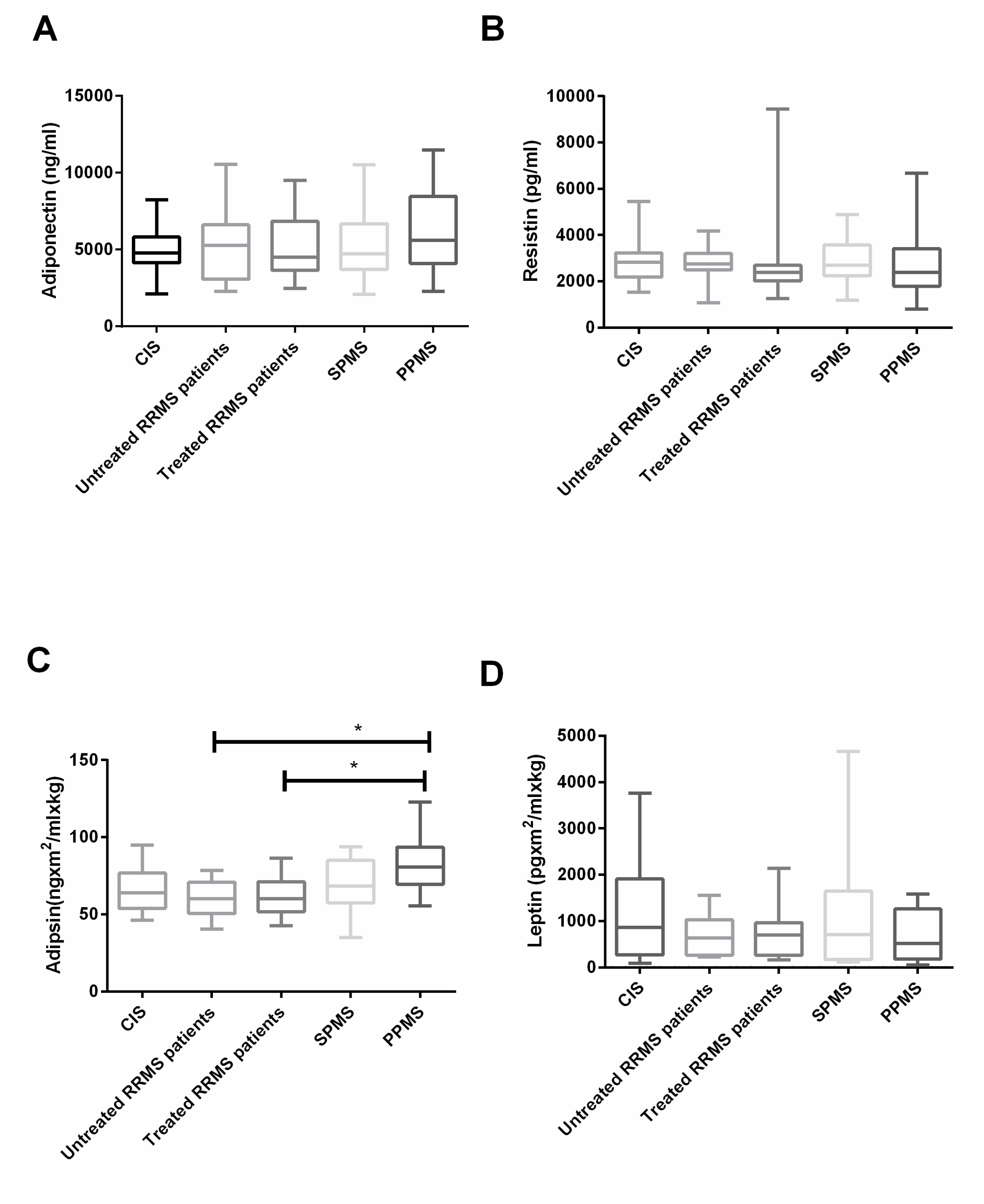

Supplement: Supplementary file 1 — Supplementary Figure 1: The levels of Adiponectin (A), Resistin (B), Adipsin (C) and Leptin (D) in MS patients including treated and untreated patients. [file 371734.f1.pdf]
